# Data on experiments result of three identical huts with shape-stabilized phase change materials in Japanese temperate climate

**DOI:** 10.1016/j.dib.2018.01.088

**Published:** 2018-02-14

**Authors:** Hyun Bae Kim, Masayuki Mae, Youngjin Choi, Takeshi Kiyota

**Affiliations:** a Department of Architecture, Graduate School of Engineering, The University of Tokyo, Japan; b JXTG Nippon Oil & Energy Corporation, Japan

## Abstract

The data in this article are the experimental results of three identical huts (Hut A, B and C), which were examined by using varying shape-stabilized PCMs (SSPCMs) sheet levels under natural and heating conditions in winter of Chiba prefecture where Japanese temperate climate. The SSPCMs sheet established the melting and solidification-temperature ranged at 19–26 °C were used. In Hut A, no SSPCM sheets were applied; in Hut B, four layers of SSPCM sheets were applied to the floor; in Hut C, one layer of SSPCM was applied to the floor, walls, and ceilings. The data provide information on the application of SSPCM sheets to improve indoor stabilization and the heating load reduction effects.

**Specifications Table**

**Table:** 

Subject area	*Energy in Buildings*
More specific subject area	*Indoor air stabilization, Heating energy reduction*
Type of data	*Excel Files*
How data was acquired	*The data are measured by thermocouples, heat flow sensor, and wattmeter in each hut.*
Data format	*.xls (Raw, analyzed)*
Experimental factors	*Comparison of indoor thermal environment according to PCM installation*
Experimental features	*Average indoor temperature, surface temperature, heat flow on each surface, heater power consumption*
Data source location	*Chiba prefecture, Japan*
Data accessibility	*Data were obtained from the experiment and accessible within this article*
Related research article	https://doi.org/10.1016/j.enbuild.2017.07.076

**Value of the data**

**Table:** 

• The data of experimental results of three identical huts using varying shape-stabilized PCMs (SSPCM) levels were examined for verifying the effect of indoor thermal condition. • In Hut A, no SSPCM sheets were applied; in Hut B, four layers of SSPCM sheets were applied to the floor; in Hut C, one layer of SSPCM was applied to the floor, walls, and ceilings. • The experimental data of the natural condition from January 14^th^ to 20^th^ (2016) shows the indoor average temperature, grove temperature, surface temperature, and heat flow in each hut. • The experimental data of the heating condition from February 3^rd^ to 9^th^ (2016) shows the average indoor temperature in each hut and the heating power consumption by a heater.

## 1 Data

**Fig. 1:**
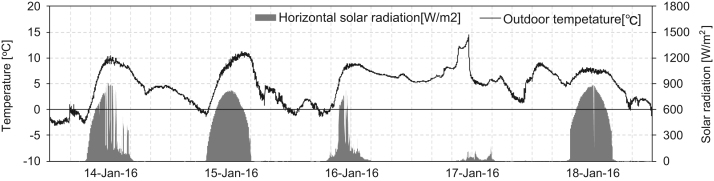
Outdoor temperature and horizontal solar radiation during January 15–19 under the natural condition.

**Fig. 2:**
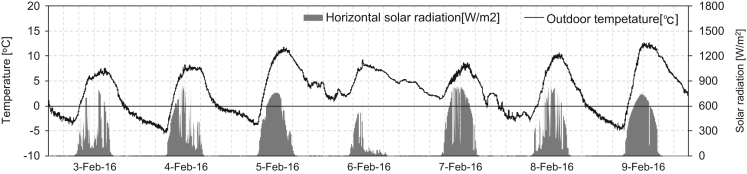
Outdoor temperature and horizontal solar radiation during February 3–9 under the heating condition.

This article data is a show of comprised of the indoor thermal environment according to SSPCM sheets different installation in three identical huts. The SSPCM sheets were made of paraffin (hexadecane and octadecane) based PCM mixed with polypropylene and elastomer to keep their shape stabilized [1]. The results of experiments were evaluated during January 15^th^–19^th^ and February 3^th^–9^th^ under both natural and heating conditions were measured every 1 minute. A weather station that measures outdoor temperature and pyrheliometers were measured horizontal solar radiation during the natural condition and heating condition as shown in Fig. 1, Fig. 2. The data sheets of natural condition show indoor air temperature, grove temperature, heat flow and the surface temperature of each surface (floor, eastern wall, ceiling). The data sheets of heating condition show indoor air temperature and power consumption by the ceramic heater.

## 2 Experimental design, materials and methods

In November 2014, three identical test huts were constructed in Chiba prefecture, Japan. To examine the effects of SSPCM sheet application and those of different SSPCM sheet application areas, no SSPCMs sheets were installed in Hut A, whereas a four-layer SSPCM sheet (6.65 MJ in total) was installed on the floor of Hut B, and one-layer SSPCM sheets were mounted on the walls, floors, and ceilings (6.74 MJ in total) of Hut C. Thermocouples were installed on each surface (five points of floor and ceiling, three points of the eastern wall) and different heights (100, 600, 1100, and 1700 mm) to measure the indoor temperature at five locations (east, west, north, south, and center). At the center point of each side, heat-flow meters (30 mm×30 mm) were placed on the SSPCM sheets to examine the amount of heat absorbed or released. The heating condition was achieved using a ceramic heater that was activated from AM 06:00 to AM 24:00 (18 hours) in every day during the measurements. The indoor heating was set at 20 °C followed by the Japanese low-energy standards [2]. Whole data mentioned above are organized into spreadsheets. Further, the layout of the thermocouples and heat-flow meters installed in the huts is presented in [1].
