# Supplementary material for: Data on experiments result of three identical huts with shape-stabilized phase change materials in Japanese temperate climate
Source: Data Brief. 2018 Feb 14;17:897–9. doi: 10.1016/j.dib.2018.01.088 (PMC5988508; doi:10.1016/j.dib.2018.01.088)
Supplement: Supplementary file 1 — Supplementary material [file mmc1.docx]

**Conflicts of interest: None**
